# Waist wins: Waist circumference tops newer indices for identifying prevalent elevated BP and hypertension in the Saudi population

**DOI:** 10.1097/MD.0000000000051392

**Published:** 2026-10-30

**Authors:** Ghadeer S. Aljuraiban, Mahmoud Aboalmeaty, Madhawi Aldhwayan, Fatma Almadani, Tagreed A. Mazi, Sara Al-Musharaf

**Affiliations:** a Department of Community Health Sciences, College of Applied Medical Sciences, King Saud University, Riyadh, Kingdom of Saudi Arabia.

**Keywords:** anthropometry, central obesity, elevated blood pressure, hypertension, visceral adiposity

## Abstract

Adiposity, commonly measured using body mass index (BMI) and waist circumference (WC), is now being assessed with newer anthropometric indices that have emerged to capture variations in body shape and height. How these indices compare in terms of discriminating prevalent elevated blood pressure (BP) and hypertension in Middle Eastern populations is unclear. This study aimed to compare the associations of WC, body roundness index (BRI), a body shape index, weight-adjusted waist index (WWI), and BMI with systolic and diastolic blood pressure (DBP) and assess how well these measures discriminate prevalent elevated BP (systolic blood pressure [SBP] 120–129 mm Hg with DBP < 80 mm Hg) and hypertension (SBP ≥ 130 or DBP ≥ 80 mm Hg). In this cross-sectional study of Saudi adults (n = 1041), anthropometric data were measured; BP was measured 3 times using standardized procedures. We ran adjusted-linear (difference in SBP/DBP per 1 standard deviation [SD] higher index) and logistic regression (odds per 1 SD). Discrimination was evaluated using receiver operating characteristic and area under the curve; optimal cutoffs were selected using Youden’s index, and sensitivity/specificity with 95% confidence intervals were obtained. In fully adjusted linear models, a 1 SD higher WC (≈17 cm) was associated with higher SBP and DBP (+4.80/+3.68 mm Hg; both *P* < .0001), with smaller differences for BMI and BRI. In fully adjusted logistic models, WC was significantly associated with elevated BP and hypertension in the pooled sample and in women, whereas elevated BP associations were not significant in men. Receiver operating characteristic analyses also showed WC had the highest discrimination for elevated BP and for hypertension. Sex-specific optimal cutoffs were identified. In Saudi adults, WC outperformed BMI, BRI, WWI, and a body shape index for discriminating prevalent elevated BP and hypertension, particularly in women. These findings support prioritizing WC in screening to identify adults more likely to have prevalent elevated BP or hypertension. Identified sex-specific cutoffs can inform clinical practice. Future prospective studies should validate these cutoffs before implementation.

## 1. Introduction

Hypertension remains a major, preventable global health issue,^[1]^ which, if untreated, can lead to cardiovascular diseases, stroke,^[2]^ kidney disease,^[3]^ metabolic syndrome, and mortality.^[4]^ According to the World Health Organization (WHO), it is estimated that about 1.28 billion adults globally (approximately 32%) had hypertension in 2019, and the prevalence is increasing.^[5]^ In Saudi Arabia, the 2019 WHO country profile reported an age-adjusted prevalence of 34% among adults aged 30 to 79, exceeding global prevalence.^[6]^

Multiple factors contribute to the development of hypertension, including behavioral, such as obesity, dietary habits, and sedentary behavior.^[7]^ In Saudi Arabia, the shift to urbanization, changes in dietary habits to a more Western-style diet characterized by high-sodium, and the increased consumption of processed foods, are among the primary causes of the increasing prevalence of hypertension.^[8]^ Because hypertension may not show noticeable symptoms, it is frequently underdiagnosed, undertreated, or poorly controlled.^[9]^ Therefore, there is a critical need for early and accurate identification of individuals at high risk of hypertension. Conventional anthropometric indices, including body mass index (BMI) and waist circumference (WC), have long been used as screening measures associated with hypertension risk,^[10]^ yet these indices are relatively insensitive to fat distribution in individuals of varying height and are limited in their ability to accurately reflect visceral adiposity.^[11]^ Systematic reviews and meta-analyses^[12,13]^ have highlighted the superiority of visceral adiposity measures over general obesity measures (i.e., BMI) for identifying cardiovascular and metabolic risk.

Excess visceral adiposity contributes to hypertension through several pathways, including activation of the renin–angiotensin–aldosterone system (RAAS), sympathetic nervous system overactivity, and chronic low-grade inflammation, which ultimately results in endothelial dysfunction.^[14]^ RAAS stimulation increases circulating angiotensin II and aldosterone, resulting in systemic vasoconstriction, water retention, and vascular remodeling, collectively leading to elevated arterial pressure.^[15]^ In addition, adiposity-induced sympathetic overactivity and cytokine-mediated endothelial nitric oxide suppression increase peripheral vascular resistance, leading to hypertension.^[16]^

Recent research in anthropometric evaluation has introduced visceral-specific anthropometric indices that may offer improved screening utility,^[17]^ namely the body roundness index (BRI),^[18]^ a body shape index (ABSI),^[19]^ and the weight-adjusted waist index (WWI).^[20]^ BRI uses height and WC, providing a more accurate assessment of visceral fat distribution. BRI has shown strong correlations with health outcomes such as cardiovascular diseases, hypertension, metabolic syndrome, and obstructive sleep apnea, and could be more clinically relevant than traditional anthropometric measures.^[21-23]^ ABSI, which incorporates WC, BMI, and height, reflects abdominal adiposity independently of BMI and has been linked to premature mortality and cardiometabolic risk.^[19]^ WWI, calculated as the ratio of WC to body weight, may also be a useful measure of central obesity and associated health risks.

Indices such as BRI, ABSI, and WWI may provide better insights into the underlying pathophysiological processes related to blood pressure (BP). However, the clinical relevance of these indices and comparisons examining their specific associations with BP remains sparse. While some longitudinal cohort studies demonstrate that BRI is strongly and positively associated with incident hypertension^[24]^ evidence for ABSI is more limited and inconsistent,^[25]^ highlighting the need for further robust investigations that directly evaluate and compare these indices. This cross-sectional study of Saudi adults aims to fill this gap by assessing the strength of relationships between these novel anthropometric indices (BRI, ABSI, WWI) and elevated BP and hypertension, and by comparing their classification performance with conventional indices (WC and BMI). The outcomes of this research have potential implications for improving hypertension screening and management, enabling targeted public health interventions, and improving clinical practice with the ultimate goal of reducing the prevalence and burden of hypertension.

## 2. Materials and methods

### 2.1. Study design

This cross-sectional study was done in Riyadh and Almadinah cities, Saudi Arabia, in 2021–2023. Recruitment invitations were sent through social media platforms, local primary care clinics, and King Saud University email lists to invite staff, students, and their families. The protocol followed the STROBE guidelines for observational research, and the study was approved by an ethics committee; the King Saud University Institutional Review Board (KSU-IRB-21-314). Each participant provided written informed consent prior to participating in the study.

This analysis represents a secondary investigation based on data collected initially to examine lifestyle factors (e.g., diet, physical activity, and stress) in relation to high BP in the Saudi population. The current study builds on that dataset to specifically address the research question of how novel anthropometric indices (BRI, ABSI, WWI) compare with conventional indices (WC and BMI) in relation to BP and hypertension among Saudi adults.

### 2.2. Participants

Eligible participants were Saudi nationals aged 18 years or older. Excluded were pregnant or lactating women, individuals with malignancy, chronic renal or hepatic failure, malabsorption syndromes, or those taking glucocorticoids or weight-loss medications (see Fig. S1, Supplemental Digital Content 1).

### 2.3. Sample-size calculation

Based on estimates from Saudi Arabia,^[6]^ *P* = .34, detecting an odds ratio of 1.30 per standard deviation (SD) rise in BRI (clinically meaningful effect size) with 80% power (β = 0.20) at a 2-sided α = 0.05, using a sample size formula for a continuous predictor in logistic regression^[26]^ required a sample of 508. Allowing for 10% attrition due to incomplete data, the target sample size became 565 participants (508/0.90). BRI was chosen for the power calculation because, at the time of study design, it was the most widely validated of the newer indices in relation to hypertension and cardiometabolic outcomes, and therefore provided the most reliable effect-size estimates to base the calculation on. The same sample size was expected to provide sufficient power for secondary comparisons with other indices such as BMI, WC, ABSI, and WWI. Because the final cohort would also support secondary comparisons involving BMI and interaction terms, a sample size of 1000 adults was deemed appropriate. Figure S1, Supplemental Digital Content 1 shows the flow of participation. A total of 1041 participants were included.

### 2.4. Collected data

Health-related data were collected by trained interviewers using a standard questionnaire. Sociodemographic characteristics, smoking status, and personal and family medical history were collected. The Arabic Global Physical Activity Questionnaire-v2^[27]^ was used to collect data on physical activity.

### 2.5. Anthropometric indices

Trained staff collected anthropometric indices in accordance with the study protocol. All measures were collected twice, and the average reading was used in the analyses. If the 2 readings differed by more than 0.5 kg for weight or 0.5 cm for other measures, a third measurement was collected, and the average of the 2 closest values was used. For weight, participants were asked to remove heavy clothing (e.g., jackets for men, abayas for women) and shoes. Weight was recorded to the nearest 0.1 kg using a multi-frequency bioelectrical impedance analyzer (InBody 570, Co. Ltd., South Korea). For height, participants were asked to stand straight without shoes. Height was recorded to the nearest 0.5 cm using a wall-mounted digital stadiometer (Seca 274, GmbH, Germany). WC was measured at the midpoint between the lowest rib and the iliac crest at the end of gentle expiration.^[28]^ A non-stretch fiberglass tape was used, and measures were recorded to the nearest 0.5 cm. For interpretation, higher values of BRI, ABSI, and WWI correspond to greater visceral adiposity and higher cardiometabolic risk, whereas lower values indicate more favorable fat distribution.

BRI was calculated using the formula by Thomas et al^[18]^: 364.2–365.5 × √{1–[WC*⁄*(2π)]^2^*⁄*[0.5 × height]^2^}.

ABSI was calculated using the formula by Krakauer and Krakauer^[19]^: WC/(BMI^2/3^ × height^1/2^).

WWI was calculated using the Park et al method^[20]^: WC/√weight.

BMI^[29]^ was calculated as: weight (kg)/height (m)^2^.

### 2.6. Blood pressure measurement

BP was measured according to the guidelines of the American College of Cardiology/American Heart Association (ACC/AHA).^[30]^ Measurements were done 3 consecutive times by trained staff, and the average reading was used. A quiet room with a comfortable room temperature was dedicated to collecting measures. Participants were asked to refrain from smoking, caffeine consumption, and exercise for 30 minutes before measurement and to empty their bladder, place their feet on the ground, and sit quietly for 3 to 5 minutes prior to measurement. The Omron BP Monitor M6 Comfort was used for all measures.

Following the ACC/AHA definitions, elevated BP was defined as a systolic blood pressure (SBP) 120 to 129 mm Hg and a diastolic blood pressure (DBP) < 80 mm Hg, while hypertension was defined as SBP ≥ 130 mm Hg or DBP ≥ 80 mm Hg.^[31]^

### 2.7. Covariates

We selected covariates a priori based on prior evidence on determinants of adiposity and BP. Sociodemographic and behavioral covariates were obtained from the standardized questionnaire and included age, sex,^[32,33]^ income, level of education,^[34]^ smoking status,^[35]^ and physical-activity level.^[36]^ We also collected family history of hypertension,^[37]^ and current use of antihypertensive medication (yes/no).^[38]^

In the fully adjusted model (model 2), we included antihypertensive medication use as a covariate to account for treatment status, because therapy can lower measured BP and may attenuate observed associations in observational analyses.^[38]^ In addition, we applied prespecified exclusion criteria to reduce confounding from conditions or medications that can affect body weight, body composition, or BP (pregnancy/lactation; malignancy; chronic renal or hepatic failure; malabsorption syndromes; glucocorticoid or weight-loss medication use).

### 2.8. Statistical analysis

All analyses were performed with SPSS version 28 (IBM Corporation, Armonk) and R version 4.3 (R Foundation for Statistical Computing, Vienna, Austria). Variable distributions were examined with the Shapiro–Wilk test. Descriptive statistics were presented as means (SD) or medians with inter-quartile ranges for continuous variables and as percentages for categorical variables.

Partial Pearson correlations adjusted for sex and age identified associations between each anthropometric index and BP. Multivariable linear regression models were used to assess changes in SBP and DBP per 1 SD increment for each index. SDs were computed in the total sample (BRI (3.3), ABSI (0.01), WWI (1.4), WC (17.3) cm, BMI (6.2) kg/m^2^) and for each sex for stratified analyses (1SD values [men = BRI 3.1; ABSI 0.01; WWI 1.4; WC 16.2; BMI 6.2]; [women = BRI 2.7; ABSI 0.01; WWI 1.3; WC 14.5; BMI 6.1]). Model 1 was adjusted for age and sex, while model 2 was adjusted for the same variables as in model 1, in addition to marital status, income, level of education, smoking status, physical activity level, family history of hypertension, and use of antihypertensive medication. Sex-stratified models used the same variables (without sex). Multivariable logistic regression analysis identified the odds of elevated BP and hypertension per 1 SD increase in each anthropometric index.

We compared the classification performance of each anthropometric index for elevated BP and hypertension by sex. Sex-specific receiver operating characteristic (ROC) curves were constructed for each anthropometric index against elevated BP and hypertension. We defined the optimal cutoff as the value that maximized Youden’s index (*J* = sensitivity + specificity − 1).^[39]^ At the selected cutoffs, sensitivity and specificity with 95% confidence intervals (CIs) were obtained using bootstrap resampling, and the area under the curve (AUC) with 95% CIs was estimated using the DeLong method.^[40]^

At these cutoffs, we calculated sensitivity and specificity along with their 95% CIs, which were obtained through bootstrap resampling with 400 iterations. The number of bootstrap replications was selected to provide a stable and reliable estimate of variability while balancing computational efficiency, consistent with established practice in applied biostatistics. The cutoffs represent the point at which each anthropometric index most accurately distinguishes individuals with elevated BP or hypertension from those without.

Discrimination was interpreted using conventional AUC cutoffs: AUC = 0.50 indicates no discrimination, 0.60 to 0.70 = poor, 0.70 to 0.80 = acceptable, 0.80 to 0.90 = excellent, and >0.90 = outstanding.^[41]^ A 2-sided *P* value below .05 was considered statistically significant.

## 3. Results

### 3.1. Baseline characteristics

This cross-sectional study included 1041 Saudi adults (397 men and 644 women) with a mean age of 31.8 years (SD = 12.2; Table 1). The majority held at least a bachelor’s degree (57.8%), and about one-third reported a monthly income above 20,000 Saudi Riyals. A notable proportion (75.5%) reported a family history of hypertension, and 14.0% were current smokers.

**Table 1:** Baseline characteristics of study participants (n = 1041).*

Parameters	Men	Women	Total
N	397	644	1041
Age (yr)	31.4 (12.4)	32.0 (12.1)	31.8 (12.2)
Moderate physical activity (min/wk)	267.9 (210.4)	245.9 (146.6)	254.2 (433.1)
Marital status			
Married	41.3	32.6	35.93
Single	57.2	56.7	56.87
Divorced	1.5	10.7	7.2
Education			
High school	28.7	27.6	28.1
Bachelor’s degree	56.2	58.9	57.8
Postgraduate degree	15.1	13.5	14.1
Income (SR/mo)			
<5000	7.3	9.2	8.5
5000 –10,000	19.4	25.2	23.0
10,000 –20,000	37.5	34.3	35.5
Above 20,000	35.8	31.4	33.0
Smoking status			
Current	28.2	5.3	14.0
Family history of hypertension	73.3	76.9	75.5
SBP	119.0 (15.2)	106.9 (14.2)	111.53 (15.7)
DBP	76.1 (11.6)	74.2 (17.1)	75.00 (15.3)
Elevated BP	15.1	4.0	8.3
Hypertension	36.8	27.0	30.7
WC (cm)	95.4 (16.2)	78.4 (14.5)	84.9 (17.3)
BMI (kg/m^2^)	27.4 (6.2)	26.4 (6.1)	26.8 (6.2)
BRI	9.8 (3.1)	6.5 (2.7)	7.8 (3.3)
ABSI	0.1 (0.01)	0.09 (0.01)	0.09 (0.01)
WWI	8.3 (1.4)	7.5 (1.3)	7.8 (1.4)

ABSI = a body shape index, AUC = area under the curve, BMI = body mass index, BP = blood pressure, BRI = body roundness index, DBP = diastolic blood pressure, SBP = systolic blood pressure, SD = standard deviation, WC = waist circumference, WWI = waist–weight index.

* Data presented as mean (SD) for continuous variables and % for frequency.

Men exhibited higher mean SBP (119.0 mm Hg vs 106.9 mm Hg) and WC (95.4 cm vs 78.4 cm), along with elevated values in novel anthropometric indices such as the BRI (9.8 vs 6.5) and WWI (8.3 vs 7.5). Overall hypertension prevalence was 30.7%, with men again showing a higher rate (36.8%) than women (27.0%).

### 3.2. Pearson partial correlation

WC consistently showed a positive linear relationship with both SBP and DBP, followed by BMI and WWI. BRI was moderately correlated, while ABSI showed only weak or no correlation with BP (Table S1, Supplemental Digital Content 2). Associations were generally stronger for SBP than DBP.

### 3.3. Association of BP with anthropometric indices

Across all indices, a higher adiposity measure was associated with higher BP (Table 2). The highest estimated difference in SBP and DBP was for WC. In model 2, a 1-SD higher WC (17 cm) was associated with a 4.80 mm Hg higher SBP (95% CI: 3.83, 5.78) and 3.68 mm Hg higher DBP (95% CI: 2.56, 4.89; both *P* < .0001). BMI was a close second, followed by BRI and WWI, with ABSI showing the smallest difference but still a statistically significant difference.

**Table 2:** Estimated SBP and DBP difference per 1 SD higher anthropometric index values (n = 1041).†

Variable	SBP	DBP
	Difference (95% CI) mm Hg	Difference (95% CI) mm Hg
BRI		
Model 1	3.94 (3.00, 4.86)*	2.20 (1.11, 3.25)*
Model 2	3.96 (3.00, 4.87)*	2.11 (1.06, 3.16)*
ABSI		
Model 1	2.42 (1.36, 3.49)*	2.34 (1.19, 3.49)*
Model 2	2.30 (1.24, 3.35)*	2.23 (1.08, 3.39)*
WWI		
Model 1	3.73 (2.89, 4.56)*	2.39 (1.45, 3.34)*
Model 2	3.74 (2.93, 4.56)*	2.35 (1.40, 3.29)*
WC		
Model 1	4.65 (3.67, 5.64)*	3.87 (2.76, 4.98)*
Model 2	4.80 (3.83, 5.78)*	3.68 (2.56, 4.89)*
BMI		
Model 1	4.00 (3.14, 4.87)*	3.08 (2.13, 4.03)*
Model 2	4.05 (3.19, 4.91)*	2.87 (1.91, 3.83)*

Model 1 was adjusted for age and sex.

Model 2 was adjusted for Model 1 + marital status, income, level of education, smoking status, physical-activity level, family history of hypertension, and antihypertensive medication use.

ABSI = a body shape index, BMI = body mass index, BP = blood pressure, BRI = body roundness index, CI = confidence interval, DBP = diastolic blood pressure, SBP = systolic blood pressure, SD = standard deviation, WC = waist circumference, WWI = waist–weight index.

* *P* < .0001.

† Mean (95% CI) differences per 1 SD of BRI (3.3), ABSI (0.01), WWI (1.4), WC (17.3) cm, BMI (6.2) kg/m^2^.

A similar pattern was observed when the sample was stratified by sex, with WC yielding the largest SBP and DBP differences per increase in SD for both men and women (Table 3). In women, WC’s association was even stronger, with over 4 mm Hg higher SBP per 1 SD increase. ABSI was significantly related to SBP and DBP in women but not in men.

**Table 3:** Estimated SBP and DBP difference per 1 SD higher anthropometric index values, stratified by sex (n = 1041).†

Variable	Men		Women	
	SBP	DBP	SBP	DBP
	Difference (95% CI) mmHg	Difference (95% CI) mmHg	Difference (95% CI) mmHg	Difference (95% CI) mmHg
BRI				
Model 2	3.77 (2.49, 5.04)**	2.23 (1.24, 3.22)**	3.28 (2.26, 4.29)**	1.62 (0.27, 2.97)**
ABSI				
Model 2	0.72 (−0.41, 1.85)	1.32 (−0.13, 2.77)	3.76 (2.31, 5.22)**	2.58 (1.50, 3.66)**
WWI				
Model 2	3.91 (2.66, 5.17)**	2.24 (1.26, 3.22)*	3.49 (2.48, 4.49)**	2.41 (1.07, 3.74)**
WC				
Model 2	4.05 (2.68, 5.43)**	2.42 (1.36, 3.49)**	4.51 (3.40, 5.63)**	3.9 (2.42, 5.38)**
BMI				
Model 2	3.95 (2.52, 5.37)**	2.44 (1.37, 3.51)**	4.27 (3.21, 5.33)**	3.29 (1.87, 4.71)**

Model 2 was adjusted for age, marital status, income, level of education, smoking status, physical-activity level, family history of hypertension, and antihypertensive medication use.

ABSI = a body shape index, BMI = body mass index, BRI = body roundness index, CI = confidence interval, DBP = diastolic blood pressure, SBP = systolic blood pressure, SD = standard deviation, WC = waist circumference, WWI = waist–weight index.

* *P* < .01.

** *P* < .0001.

† Mean (95% CI) differences per 1 SD of [(men: BRI (3.1), ABSI (0.01), WWI (1.4), WC (16.2) cm, BMI (6.2) kg/m^2^); (women: BRI (2.7), ABSI (0.01), WWI (1.3), WC (14.5) cm, BMI (6.1) kg/m^2^)].

### 3.4. Odds of elevated BP and hypertension

In the fully adjusted model 2, all anthropometric indices, except ABSI and BMI, were significantly associated with elevated BP in the total sample (Table 4). These associations were consistently stronger in women (Table 5). However, in men, associations with BRI, ABSI, WWI, WC, and BMI were not significant.

**Table 4:** Odds of elevated BP and hypertension per 1 SD increase in each anthropometric index (n = 1041).*

Index	Model 1		Model 2	
	OR (95% CI)	*P*-value	OR (95% CI)	*P*-value
Elevated BP				
BRI	1.36 (1.12, 1.66)	.002	1.41 (1.53, 1.74)	.0009
ABSI	1.07 (0.80, 1.42)	.66	1.08 (0.81, 1.44)	.62
WWI	1.29 (1.04, 1.61)	.02	1.33 (1.06, 1.67)	.01
WC	1.21 (0.93, 1.57)	.15	1.33 (1.02, 1.74)	.04
BMI	1.15 (0.92, 1.45)	.21	1.24 (0.98, 1.56)	.07
Hypertension				
BRI	1.68 (1.40, 2.03)	<.0001	1.68 (1.40, 2.02)	<.0001
ABSI	0.78 (0.65, 1.94)	.49	0.78 (0.65, 1.96)	.49
WWI	1.65 (1.42, 1.93)	<.0001	1.65 (1.41, 1.92)	<.0001
WC	2.05 (1.70, 2.47)	<.0001	2.02 (1.67, 2.44)	<.0001
BMI	1.82 (1.56, 2.12)	<.0001	1.78 (1.52, 2.09)	<.0001

Model 1 was adjusted for age and sex. Model 2 was adjusted for model 1 + marital status, income, level of education, smoking status, physical-activity level, family history of hypertension, and antihypertensive medication use.

ABSI = a body shape index, BMI = body mass index, BP = blood pressure, BRI = body roundness index, CI = confidence interval, OR = odds ratio, SD = standard deviation, WC = waist circumference WWI = waist–weight index.

* Odds (95% CI) of elevated BP and hypertension per 1 SD of BRI (3.3), ABSI (0.01), WWI (1.4), WC (17.3) cm, BMI (6.2) kg/m^2^.

**Table 5:** Odds of elevated BP and hypertension per 1 SD increase in each index, stratified by sex (n = 1041).†

Index	Elevated BP		Hypertension	
	Men	Women	Men	Women
	OR (95% CI)	OR (95% CI)	OR (95% CI)	OR (95% CI)
BRI	1.06 (0.79, 1.42)	2.23 (1.37, 3.65)*	1.52 (1.19, 1.93)*	2.04 (1.52, 2.74)**
ABSI	1.12 (0.78, 1.60)	1.05 (0.64, 1.72)	0.76 (0.57, 1.03)	0.78 (0.62, 0.98)
WWI	1.10 (0.83, 1.45)	1.88 (1.29, 2.74)*	1.47 (1.17, 1.85)*	1.86 (1.50, 2.31)**
WC	1.06 (0.77, 1.47)	2.30 (1.42, 3.74)*	1.68 (1.28, 2.20)*	2.53 (1.91, 3.35)**
BMI	0.99 (0.73, 1.33)	1.83 (1.25, 2.68)*	1.57 (1.24, 2.00)*	2.03 (1.63, 2.53)**

Model 2 was adjusted for age, marital status, income, level of education, smoking status, physical-activity level, family history of hypertension, and antihypertensive medication use.

ABSI = a body shape index, BMI = body mass index, BP = blood pressure, BRI = body roundness index, CI = confidence interval, OR = odds ratio, SD = standard deviation, WC = waist circumference WWI = waist–weight index.

* *P* < .01.

** *P* < .0001.

† Odds (95% CI) of elevated BP and hypertension per 1 SD of [(men: BRI (3.1), ABSI (0.01), WWI (1.4), WC (16.2) cm, BMI (6.2) kg/m^2^); (women: BRI (2.7), ABSI (0.01), WWI (1.3), WC (14.5) cm, BMI (6.1) kg/m^2^)].

In the fully adjusted model 2, all anthropometric indices, except ABSI, were significantly associated with increased odds of hypertension (Table 4). Similar to elevated BP, these associations were consistently stronger in women (Table 5). In men, significant associations also persisted, particularly for BRI, WC, and WWI.

### 3.5. Discrimination and optimal cutoffs

The discrimination of the 5 anthropometric indices for identifying prevalent elevated BP and hypertension is presented in Table S2, Supplemental Digital Content 3.

For elevated BP in men, discrimination was weak for all indices, with AUCs ranging from 0.46 (ABSI) to 0.57 (WWI). BRI (AUC = 0.56) and WWI (AUC = 0.57) showed slightly better performance than BMI (AUC = 0.51) and WC (AUC = 0.49). However, none demonstrated strong discrimination (Table 6 and Fig. 1). At the optimal cutoffs (Table 6), men’s sensitivities ranged from 0.22 to 0.59 and specificities from 0.52 to 0.85, with BRI providing the highest sensitivity (0.59) and WC the highest specificity (0.85). Among women with elevated BP, WC yielded the highest AUC (0.71), closely followed by BRI and BMI (both 0.70) and WWI (0.68). ABSI performed poorly in both sexes (AUC ≤ 0.51; Table 6 and Fig. 1). In women, sensitivities were generally higher (0.14–0.78): BMI had the highest sensitivity (0.78) with moderate specificity (0.57); ABSI had very high specificity (0.97) but low sensitivity (0.14); and WC/BRI showed more balanced profiles (sensitivities 0.62–0.59; specificities both 0.75; Table 6).

**Table 6:** AUC and optimal cutoff points of anthropometric indices in relation to elevated BP and hypertension stratified by sex.

Predictor	Men				Women			
	AUC (95% CI)	Cutoff	Sensitivity	Specificity	AUC (95% CI)	Cutoff	Sensitivity	Specificity
Elevated BP								
BRI	0.56 (0.49, 0.63)	9.67	0.59	0.52	0.70 (0.60, 0.79)	7.58	0.59	0.75
ABSI	0.45 (0.38, 0.52)	0.11	0.22	0.80	0.42 (0.31, 0.54)	0.08	0.14	0.97
WWI	0.57 (0.50, 0.64)	9.05	0.41	0.76	0.68 (0.58, 0.78)	7.88	0.65	0.67
WC	0.49 (0.42, 0.56)	112.0	0.22	0.85	0.71 (0.60, 0.78)	78.50	0.62	0.75
BMI	0.51 (0.44, 0.58)	31.32	0.31	0.78	0.70 (0.60, 0.79)	26.46	0.78	0.57
Hypertension								
BRI	0.65 (0.59, 0.70)	11.12	0.47	0.81	0.73 (0.69, 0.78)	6.68	0.68	0.71
ABSI	0.46 (0.41, 0.52)	0.08	0.67	0.55	0.46 (0.41, 0.51)	0.08	0.03	0.98
WWI	0.64 (0.58, 0.69)	9.02	0.43	0.83	0.71 (0.66, 0.75)	7.67	0.65	0.69
WC	0.71 (0.65, 0.75)	99.50	0.56	0.75	0.76 (0.72, 0.81)	79.50	0.78	0.69
BMI	0.69 (0.64, 0.75)	26.33	0.72	0.58	0.76 (0.72, 0.80)	26.50	0.75	0.68

ABSI = a body shape index, AUC = Area under the curve, BMI = body mass index, BP = blood pressure, BRI = body roundness index, CI = confidence interval, WC = waist circumference, WWI = waist–weight index.

**Figure 1.**
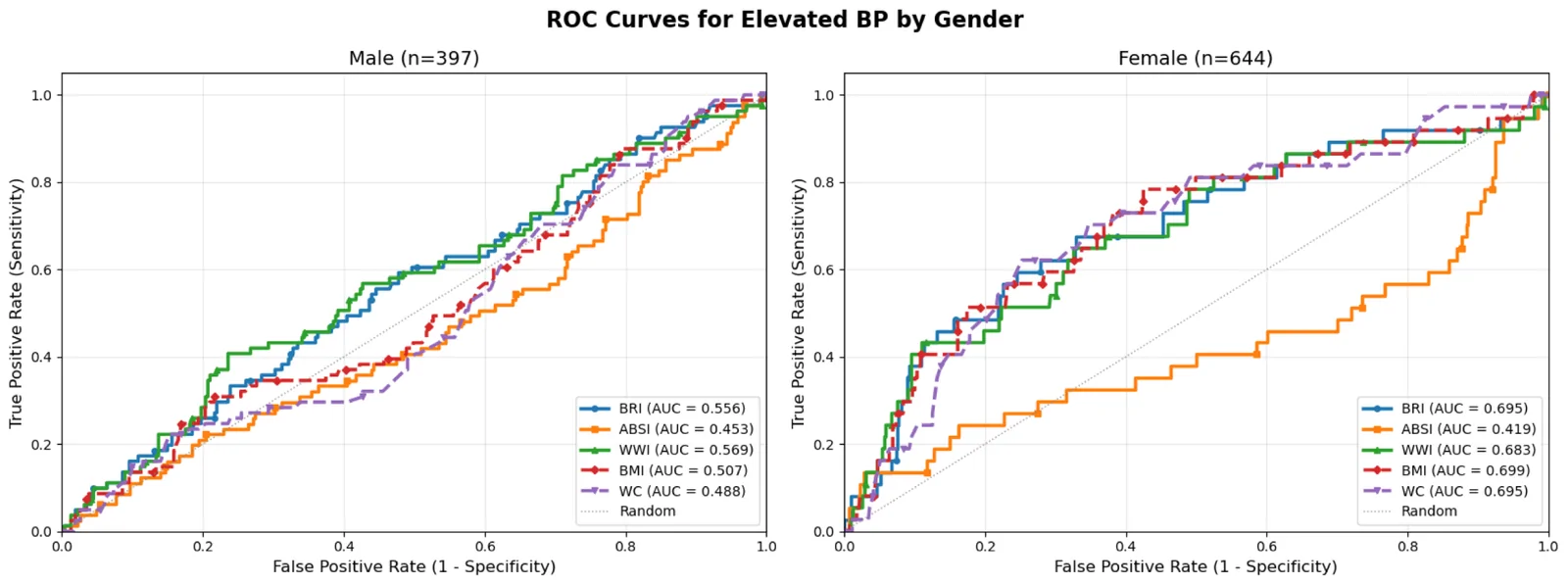
Receiver operating characteristic (ROC) curves and area under the curve (AUC) for body roundness index (BRI), a body shape index (ABSI), waist–weight index (WWI), waist circumference (WC), and body mass index (BMI) for identifying prevalent elevated blood pressure in males (left) and females (right). ABSI = a body shape index, AUC = area under the curve, BMI = body mass index, BRI = body roundness index, ROC = receiver operating characteristic, WC = waist circumference, WWI = waist–weight index.

In men, discrimination for hypertension improved across all indices compared to elevated BP. WC demonstrated the highest discrimination (AUC = 0.71), followed by BMI (AUC = 0.69) and BRI (AUC = 0.65). WWI (AUC = 0.64) outperformed ABSI (AUC = 0.61), which remained the weakest measure (Table 6 and Fig. 2). At the optimal cutoffs, BMI showed the highest sensitivity (0.72), WWI the highest specificity (0.83), and WC offered a balanced profile (sensitivity 0.56; specificity 0.75; Table 6). In women with hypertension, both WC (AUC = 0.76) and BMI (AUC = 0.76) had the strongest performance, followed by BRI (0.73) and WWI (0.71). ABSI again showed the lowest discrimination (AUC = 0.61; Table 6 and Fig. 2). Sensitivity and specificity patterns paralleled those for elevated BP: WC and BMI had the highest sensitivities (0.78 and 0.75, respectively) with specificities around 0.68 to 0.69, whereas ABSI had very high specificity (0.98) but negligible sensitivity (0.03; Table 6).

**Figure 2.**
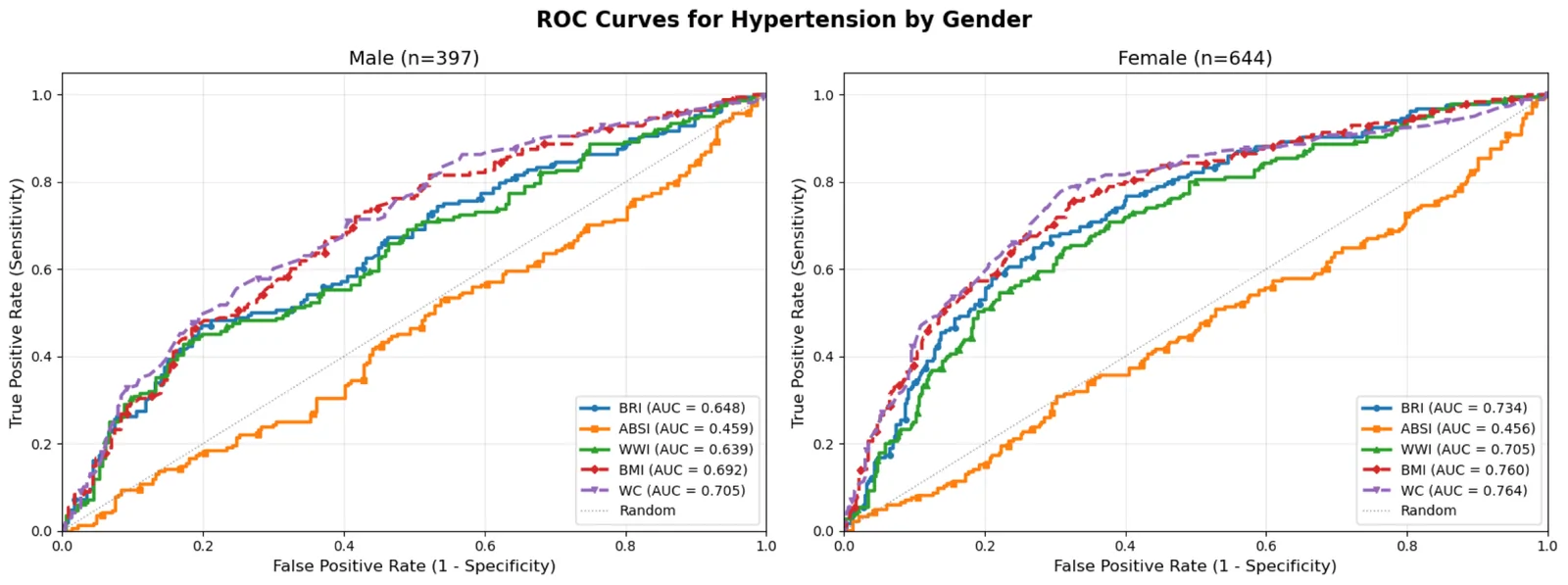
Receiver operating characteristic (ROC) curves and area under the curve (AUC) for body roundness index (BRI), a body shape index (ABSI), waist–weight index (WWI), waist circumference (WC), and body mass index (BMI) for identifying prevalent hypertension in males (left) and females (right). ABSI = a body shape index, AUC = area under the curve, BMI = body mass index, BRI = body roundness index, ROC = receiver operating characteristic, WC = waist circumference, WWI = waist–weight index.

For men, the cutoffs were:

- Elevated BP: BRI 9.7, ABSI 0.11, WWI 9.05, WC 112.0 cm, BMI 31.32 kg/m^2^
- Hypertension: BRI 11.1, ABSI 0.08, WWI 9.02, WC 99.5 cm, BMI 26.33 kg/m^2^

For women, the cutoffs were:

- Elevated BP: BRI 7.6, ABSI 0.08, WWI 7.9, WC 78.5 cm, BMI 26.5 kg/m^2^
- Hypertension: BRI 6.7, ABSI 0.08, WWI 7.7, WC 79.5 cm, BMI 26.5 kg/m^2^ (Table 6).

## 4. Discussion

### 4.1. Main findings

In this cross-sectional study, the newer anthropometric indices (BRI, ABSI, WWI) did not outperform the “traditional” measures of WC and BMI in either their associations with BP or their ability to discriminate elevated BP and hypertension. In Saudi adults, visceral adiposity (proxied by these indices) was positively related to BP, with the strongest linear associations for WC and then BMI. When associations were standardized (i.e., expressed per 1-SD higher value of each index), WC showed the largest BP differences, followed by BMI and BRI. WWI and ABSI were smaller, and ABSI was significant in the pooled sample and in women but not in men.

Anthropometric indices varied in their ability to discriminate prevalent elevated BP and hypertension. AUC-based discrimination was modest for elevated BP, especially in men, and considerably stronger for hypertension, with WC and BMI being the best-performing single measures in both sexes. At optimal cutoffs, WC and BMI provided the most balanced sensitivity/specificity profiles, whereas ABSI had very high specificity but low sensitivity (e.g., women: specificity 0.97, sensitivity 0.14). These findings support the routine use of WC (and BMI where WC is not feasible) for BP screening and case identification in Saudi adults, consistent with WHO^[28]^ and AHA^[42]^ guidelines, while indicating that newer indices add little beyond these traditional measures in this cross-sectional setting.

### 4.2. Comparison with other studies

In fully adjusted linear models, a 1-SD higher WC was associated with the largest differences in SBP and DBP. This is consistent with WC’s close relationship to visceral adiposity and its downstream hemodynamic and inflammatory effects (e.g., RAAS activation, sympathetic overactivity, endothelial dysfunction).^[14-16]^

Our results agree with the findings from large cohort studies that show that compared to general obesity, abdominal adiposity has a stronger association with prehypertension, as demonstrated by the added value of WC over and above BMI.^[43,44]^ In the present data, WC showed the largest effect sizes and the highest AUCs. Central obesity can lead to higher BP through mechanisms involving sympathetic nervous system activation, RAAS signaling within adipose tissue, insulin resistance, endothelial dysfunction, and sodium retention.^[14-16]^ Aligning with our observations, these mechanisms^[45]^ have repeatedly been shown to be involved in obesity-related hypertension and are stronger for visceral than peripheral adiposity.^[14]^

For the newer indices, our findings are broadly consistent with prior work. BRI generally showed discrimination in our study and has been reported elsewhere to perform comparably to BMI but below the WC/waist-to-height ratio.^[46]^ In contrast, ABSI underperformed for hypertension and, in other studies, other cardiometabolic endpoints.^[25]^ This may be a consequence of the calculation used to determine ABSI, which excludes the mass and height components from waist size (a “shape residual”), thereby attenuating the central adiposity signal that most strongly tracks with BP physiology.^[47]^ Accordingly, ABSI was reported to have a weaker association with incident or prevalent hypertension than WC, BMI, or BRI, and that ABSI performs better for mortality than for chronic cardiometabolic disease endpoints.^[47]^ Evidence for WWI is still relatively scarce and recent; population studies link higher WWI to greater hypertension risk, particularly in men, which is also consistent with our sex-stratified estimates showing WWI had higher specificity but low sensitivity in men.^[48,49]^ The sensitivity/specificity profiles in our study imply different use-cases: indices with higher sensitivity (e.g., BMI in men, WC in women) are better for first-line screening, whereas indices with higher specificity (e.g., WWI in men) are better for “rule-in” confirmation. From a population-level perspective, excess weight, especially visceral, is responsible for a large percentage of the hypertension burden.^[50]^ Consistent with that, waist-based measures (particularly WC) tend to discriminate hypertension well; however, in our data, WWI performed moderately and did not surpass WC or BMI.

Another important consideration is the role of family history of hypertension. Genetic predisposition is a well-established determinant of BP^[51]^; however, individuals without a family history may not be routinely flagged for early screening or preventive interventions. In this context, anthropometric indices such as BRI, ABSI, and WWI could serve as valuable tools to identify at-risk individuals who might otherwise be overlooked. Previous studies have shown that the contribution of obesity and central adiposity to hypertension risk is substantial even in those without a familial predisposition, highlighting the importance of body-shape–based indices in broadening risk stratification beyond genetic factors.^[46,52]^ This suggests that the utility of these indices extends to populations with and without family history, making them relevant for more comprehensive hypertension screening.

### 4.3. Gender differences

Women showed larger per-SD BP differences and higher odds ratios for BP associated with several indices, and WC was the dominant anthropometric measure. Previous studies^[53,54]^ showed sex disparities in the relationship between central adiposity and hypertension, as well as the joint impact of BMI and WC, which can be especially pronounced at higher adiposity levels. The stronger results for WC align with evidence that central adiposity exerts stronger BP effects in women and that, at a given waist level, women may experience greater BP consequences, attributed to sex differences in visceral fat distribution and BP responses.^[55,56]^ Consistent with this literature, an equivalent WC may indicate relatively greater BP risk in women, helping explain the stronger WC–BP relationship we observed.

For men with elevated BP, none of the 5 indices (BRI, ABSI, WWI, WC, BMI) showed significant associations after full adjustment. The elevated BP phenotype (SBP 120–129 mm Hg with DBP < 80 mm Hg) is a narrow, transitional phenotype with fewer events and smaller effect sizes than hypertension. In sex-stratified, fully adjusted models, this reduces statistical power and widens CIs, so nonsignificant estimates should be interpreted as possibly underpowered rather than null.^[57]^ Our gender-specific cutoffs (lower in women) align with these established patterns. In previously published studies from Saudi Arabia, WC cutoffs ranged from 92 cm (for men) and 87 cm (for women) for metabolic-syndrome risk^[58]^ to 97 cm (for men) and 90 cm (for women) for hypertension in a national survey.^[59]^ Our WC cutoff for hypertension in men was higher (99.5 cm) than for women (79.5 cm). Overall, these sex-specific cutoffs support using different WC triggers for referral and follow-up in women versus men.

### 4.4. Clinical and public-health implications

Our findings suggest that measuring WC is practical, low-cost, and, within this cohort, was most informative for identifying adults more likely to have prevalent elevated BP or hypertension. In practice, these cutoffs can be implemented by primary-care clinicians, nurses, and community health workers as part of intake or outreach screening, with sex-specific cutoffs used to triage patients for confirmatory BP checks, lifestyle counseling, or follow-up. This is in agreement with WHO and ACC/AHA recommendations to incorporate WC into risk assessment. Even though our conclusions align with existing guidance, the study adds locally relevant evidence by providing comparisons of 5 indices (WC, BMI, BRI, ABSI, WWI) in Saudi adults, identifying sex-specific optimal cutoffs linked to sensitivity/specificity, and using contemporary ACC/AHA categories that include “elevated BP,” which most prior Saudi work did not examine.

Our gender-specific, data-driven cutoffs can complement these guidelines and may be valuable for refining screening strategies in Middle Eastern populations. Our ROC cutoffs are not directly comparable with previous Saudi reports because prior work defined “high BP” using previous thresholds (≥130/85 mm Hg) for the metabolic-syndrome component and for hypertension (≥140/90 mm Hg), and did not identify an “elevated BP” category. These definitional differences help explain between-study discrepancies (e.g., our lower female WC cutoff for hypertension). For BMI, our hypertension cutoffs (~26–26.5 kg/m^2^) are broadly consistent with Saudi studies^[58]^ that link BMI in the high-20s to cardiometabolic risk (e.g., metabolic syndrome and hypertension outcomes), placing our values within or slightly below those ranges. To our knowledge, comparable Saudi cutoffs for BRI, WWI, and ABSI in relation to BP have not been reported, highlighting the need for local validation and calibration of these indices before routine use.

Anthropometry measures should complement BP measurement and broader risk profiling, especially in high-volume clinics. BP is a measure that varies within individuals and can be misclassified on a single reading; anthropometric indices capture a stable adiposity signal that helps flag people with normal/borderline BP who nonetheless have elevated risk. In busy settings, a quick WC screen can determine who needs repeat BP checks, ambulatory monitoring, or early preventive counseling. WC provides the best single anthropometric screen, while BMI could be an acceptable alternative, based on the performance we observed, if waist measurement is not feasible.

### 4.5. Strengths and limitations

Strengths of this study include a relatively large sample size, standardizing associations “per 1-SD” to place all indices on a common scale, and repeated measures of variables of interest following standard procedures. Furthermore, multivariable adjustment for sociodemographic and behavioral factors (age, sex, marital status, education, income, smoking, physical activity, family history, antihypertensive medication use) reduces confounding and supports the robustness of the observed WC/BMI associations; that these associations persisted after adjustment increases confidence that they are not solely explained by lifestyle or socioeconomic differences.

Limitations include the cross-sectional design. We measured anthropometry and BP at the same visit, so we could not establish temporality or support causal inference. Reverse causation is possible because participants with diagnosed hypertension may have changed diet, physical activity, or weight, and antihypertensive treatment can lower measured BP. Our ROC analyses quantify discrimination of prevalent elevated BP and hypertension, not future BP change or incident hypertension.

In addition, we recruited participants from only 2 cities (Riyadh and Almadinah). Generalizability is further limited by sample composition and recruitment, by which women were overrepresented (62%), the mean age was relatively young (31.8 years), and participants were volunteers recruited through clinics, social media, and a university setting; groups that may differ from the broader Saudi population in education, income, health behaviors, and access to health care. These features likely limit extrapolation to older adults, rural areas, and men, and should be considered when applying cutoffs to national programs.

The relatively small sample of those with elevated BP may have reduced statistical power and precision for that transitional phenotype, especially in sex-stratified models; consequently, nonsignificant findings in men for elevated BP should be interpreted cautiously as potential type-II (underpowered) results rather than as evidence of no association. Additionally, lifestyle variables were self-reported, which introduces potential misclassification. Residual confounding may persist despite adjustment (e.g., antihypertensive medication use) and lack of external validation of the sex-specific ROC cutoffs; these thresholds were derived internally (Youden’s index) and require confirmation in independent Saudi/Middle Eastern cohorts before implementation. Finally, generalizability to other populations and ethnic groups requires confirmation because optimal anthropometric cutoffs are known to vary by ancestry, sex, and age, and can be influenced by measurement protocols and background risk factors.

## 5. Conclusions

In this cross-sectional analysis, WC showed the strongest associations with BP and the highest discrimination for elevated BP in women and for hypertension in men. WC is practical for routine screening to flag adults more likely to have prevalent elevated BP or hypertension. The sex-specific cutoffs derived here reflect internal thresholds for identifying prevalent elevated BP and hypertension in this sample. Future research should validate these cutoffs in independent and diverse populations and evaluate longitudinal performance for incident hypertension and long-term BP trajectories. Studies should also assess whether combining anthropometric indices with cardiometabolic biomarkers, such as lipid profiles and resting heart rate, improves risk stratification beyond anthropometry alone.

## Author contributions

**Conceptualization:** Ghadeer S. Aljuraiban, Mahmoud Aboalmeaty, Madhawi Aldhwayan, Tagreed A. Mazi.

**Data curation:** Ghadeer S. Aljuraiban, Fatma Almadani.

**Formal analysis:** Ghadeer S. Aljuraiban, Mahmoud Aboalmeaty, Madhawi Aldhwayan, Fatma Almadani, Tagreed A. Mazi.

**Funding acquisition:** Madhawi Aldhwayan, Sara Al-Musharaf.

**Investigation:** Mahmoud Aboalmeaty, Fatma Almadani.

**Methodology:** Ghadeer S. Aljuraiban, Mahmoud Aboalmeaty.

**Resources:** Ghadeer S. Aljuraiban.

**Supervision:** Ghadeer S. Aljuraiban, Tagreed A. Mazi, Sara Al-Musharaf.

**Writing – original draft:** Ghadeer S. Aljuraiban.

**Writing – review & editing:** Mahmoud Aboalmeaty, Tagreed A. Mazi, Sara Al-Musharaf.






