# Supplementary material for: Waist wins: Waist circumference tops newer indices for identifying prevalent elevated BP and hypertension in the Saudi population
Source: Medicine (Baltimore). 2026 Oct 30;105(44):e51392. doi: 10.1097/MD.0000000000051392 (PMC13727258; doi:10.1097/MD.0000000000051392)
Supplement: Supplementary file 1 [file medi-105-e51392-s001.docx]

**Figure 1. Flow Diagram**

Assessed for eligibility (n=1,204)

## Enrollment

Excluded (n=163):

- Did not complete questionnaires and/or body measurements (n=28)
- Pregnant or lactating women (n=4)
- Used nutritional supplements of minerals and vitamins, antihypertensive medications, corticosteroid medications, antidepressant medications, hormone therapy (n=14)
- Had a previous history of cardiovascular disease, stroke, T1DM, or dyslipidemia (n=56)
- Energy consumption below 500 kcal/d and above 6,000 kcal/d (n=26)
- Withdrew (n=35)

Included (n= 1,041)
