# Supplementary material for: Waist wins: Waist circumference tops newer indices for identifying prevalent elevated BP and hypertension in the Saudi population
Source: Medicine (Baltimore). 2026 Oct 30;105(44):e51392. doi: 10.1097/MD.0000000000051392 (PMC13727258; doi:10.1097/MD.0000000000051392)
Supplement: Supplementary file 2 [file medi-105-e51392-s002.docx]

| Supplementary Table 1. Pearson partial correlation between SBP, DBP and anthropometric indices (n=1,041)^a^ | | | | | | | | | | |
| --- | --- | --- | --- | --- | --- | --- | --- | --- | --- | --- |
|  | **BRI** | **P-value** | **ABSI** | **P-value** | **WWI** | **P-value** | **BMI** | **P-value** | **WC** | **P-value** |
| SBP | 0.25 | <0.0001 | 0.07 | 0.03 | 0.26 | <0.0001 | 0.27 | <0.0001 | 0.28 | <0.0001 |
| DBP | 0.13 | <0.0001 | 0.05 | 0.14 | 0.15 | <0.0001 | 0.19 | <0.0001 | 0.21 | <0.0001 |
| ^a^ Data presented as Pearson Correlation Coefficient (R); P< 0.05 considered significant.  Abbreviations: Systolic blood pressure (SBP), diastolic blood pressure (DBP), body roundness index (BRI), A body shape index (ABSI), waist–weight index (WWI), waist circumference (WC), and body mass index (BMI) in predicting elevated blood pressure. | | | | | | | | | | |
