# Supplementary material for: Waist wins: Waist circumference tops newer indices for identifying prevalent elevated BP and hypertension in the Saudi population
Source: Medicine (Baltimore). 2026 Oct 30;105(44):e51392. doi: 10.1097/MD.0000000000051392 (PMC13727258; doi:10.1097/MD.0000000000051392)
Supplement: Supplementary file 3 [file medi-105-e51392-s003.docx]

| **Supplementary Table 2. Discriminative performance of anthropometric indices for elevated BP and hypertension (n=1,041)** | | |
| --- | --- | --- |
| **Predictor** | **Elevated BP** | **Hypertension** |
|  | AUC (95% CI) | AUC (95% CI) |
| BRI | 0.70 (0.65, 0.75) | 0.70 (0.67, 0.74) |
| ABSI | 0.62 (0.57, 0.68) | 0.64 (0.60, 0.67) |
| WWI | 0.66 (0.61, 0.71) | 0.69 (0.66, 0.74) |
| WC | 0.66 (0.61, 0.71) | 0.75 (0.72, 0.78) |
| BMI | 0.59 (0.54, 0.65) | 0.74 (0.70, 0.77) |
| Abbreviations: Area under the curve (AUC), blood pressure (BP), body roundness index (BRI), A body shape index (ABSI), waist–weight index (WWI), waist circumference (WC), and body mass index (BMI) in predicting elevated blood pressure. | | |
